# Mosquito-larvicidal Binary (BinA/B) proteins for mosquito control programs —advancements, challenges, and possibilities

**DOI:** 10.1016/j.cris.2021.100028

**Published:** 2021-12-18

**Authors:** Mahima Sharma, Vinay Kumar

**Affiliations:** 1 Radiation Biology & Health Sciences Division, Bhabha Atomic Research Centre, Mumbai, Maharashtra 400085, India

**Keywords:** *Lysinibacillus sphaericus*, Binary (BinAB) toxin, Cqm1 receptor, BinAB internalization, Receptor-mediated pore formation, BinAB cytotoxicity, Bioremediation, Cancer therapeutics

## Abstract

The increasing global burden of mosquito-borne diseases require targeted, environmentally friendly, and sustainable approaches for effective vector control without endangering the non-target beneficial insect population. Biological interventions such as biopesticides, Wolbachia-mediated biological controls, or sterile insect techniques are used worldwide. Here we review Binary or BinAB toxin—the mosquito-larvicidal component of WHO-recognized *Lysinibacillus sphaericus* bacterium employed in mosquito control programs. Binary (BinAB) toxin is primarily responsible for the larvicidal effect of the bacterium. BinAB is a single-receptor-specific toxin and is effective against larvae of Culex and Anopheles, but not against *Aedes aegypti*. The receptor in Culex, the Cqm1 protein, has been extensively studied. It is a GPI-anchored amylomaltase and is located apically in the lipid rafts of the larval-midgut epithelium. The interaction of the toxin components with the receptor is crucial for the mosquito larvicidal activity of the BinAB toxin. Here we extend support for the pore formation model of BinAB toxin internalization and the role of toxin-glycan interactions in the endoplasmic reticulum in mediating larval death. BinAB is phylogenetically safe for humans, as Cqm1-like protein is not expected in the human proteome. This review aims to initiate targeted R&D efforts, such as applying fusion technologies (chimera of BinA, chemical modification of BinA), for efficient mosquito control interventions. In addition, the review also examines other areas such as bioremediation and cancer therapeutics, in which *L. sphaericus* is proving useful and showing potential for further development.

## Highlights

• Binary (BinAB) toxin is primarily responsible for the larvicidal action of the WHO recognized mosquito-larvicidal bacterium *Lysinibacillus sphaericus*.
• BinAB is a single receptor-specific toxin, active against larvae of Culex and Anopheles, but not *Aedes aegypti*.
• The target receptor in Culex is Cqm1 protein, a GPI-anchored amylomaltase located apically in the lipid-rafts of the larval-midgut epithelium.
• Interaction of the toxin components with the receptor is critical for the larvicidal activity of the toxin.
• Evidences support the pore formation model for BinAB toxin internalization and the role of toxin-glycan interactions in the endoplasmic reticulum in mediating larval death.
• Targeted R&D efforts are required to maintain the sustainability and improve efficacy of the eco-friendly BinAB proteins for efficient mosquito control interventions.

## Graphical abstract

**Figure:**
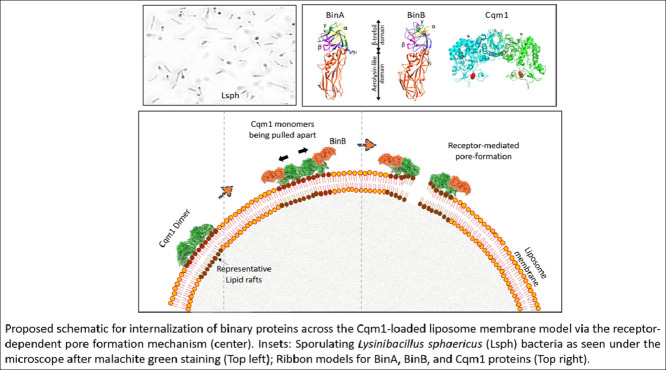

## Overview

Mosquitoes are the most common vectors known for transmitting deadly diseases such as dengue, filariasis, Japanese encephalitis**,** malaria, West Nile fever, and Zika. The recurrence of existing mosquito-borne diseases and the emergence of new diseases leads to delirious effects on human lives. In the absence of any effective vaccine or drug, prevention and control of these debilitating diseases depends entirely on effective vector control. As with personal protective shields and mosquito repellant sprays, the available physical and chemical approaches are limited by their long-term ineffectiveness, environmental incompatibility, and development of resistance. Biocontrol agents, such as viruses, fungi, bacteria, invertebrate predators, and fishes, offer greener and safer approaches.

*Culex quinquefasciatus* is a mosquito species widespread on all continents and in different ecological zones (Bhattacharya, Basu and Sajal, 2016). Two of the primary pathogens transmitted by this mosquito are West Nile Virus and the Japanese encephalitis virus, which threaten public health worldwide (Daep, Muñoz-Jordán and Eugenin, 2014). Biocontrol agents used to control Culex include entomopathogenic bacteria such as *Bacillus thuringiensis subspecies israelensis* (Bti) and *Lysinibacillus sphaericus* (Lsph, formerly known as *Bacillus sphaericus*), which have attracted worldwide attention over the past few decades. These bacteria produce proteins during sporulation which display significant mosquito-larvicidal activity. Their commercial formulations, VectoBac (Bti) (Russell et al., 2003) and VectoLex (Lsph) (Brown et al., 2004), have proven to be effective and are consistently used in mosquito control programs (Lacey, 2007). Bti produces several proteins that accumulate in crystals, with insecticidal effects against various orders, including Lepidoptera, Coleoptera, and Diptera (Valtierra-de-Luis et al., 2020). The Cry toxins from Bti could also kill human cancer cells by interacting with specific receptors (Mendoza-Almanza et al., 2020). In the case of Lsph, only a few strains are toxic to mosquitoes. Some of the known larvicidal Lsph strains include 1593, 2297, and 2362, respectively (Berry, 2012). During the vegetative phase, pathogenic Lsph strains produce many insecticidal proteins, including Mtx, Cry, and Sphaericolysin, *albeit* with low virulence (Berry, 2012). The binary or BinAB proteins, produced during sporulation as parasporal crystalline inclusions, are primarily responsible for the larvicidal action of Lsph. It targets Culex (very susceptible), Anopheles (moderately susceptible), and a few species of Aedes (susceptible— little or none) but not *Aedes aegypti* (Berry, 2012). A recent review extensively covers the earlier literature and details of the toxin components of Bti and Lsph and their resistance mechanism (Silva-Filha et al., 2021).

The use of Bti for mosquito control programs remains divisive, despite extensive use world-wide. A recent detailed review by Bruhl *et al*. (Brühl et al., 2020) alerts on the undesired effects of Bti on non-target organisms, on the environment, and the food chain. In contrast, Lsph has been shown to be highly specific, with no adverse effects on non-target organisms such as honeybees, other mosquito predators, chironomids, and other eukaryotic organisms, and is environmentally friendly (Brown et al., 2004, Lacey and Mulla, 1990, Lacey and Siegel, 2000). The World Health Organization (WHO) has examined Lsph for its effectiveness in combating mosquitoes (World Health Organization OMDLS, 1985). With an LC_90_ value of 0.024 ppm, Lsph shows higher toxicity against *Culex quinquefasciatus* than Bti with the LC_90_ value of 0.057 ppm (Zahiri, Federici and Mulla, 2004). In addition, Lsph strains have proven to be relatively more effective in polluted water and show a more prolonged residual activity due to longer persistence or recycling (Lacey, 2007, Berry et al., 1987).

**Figure 1:**
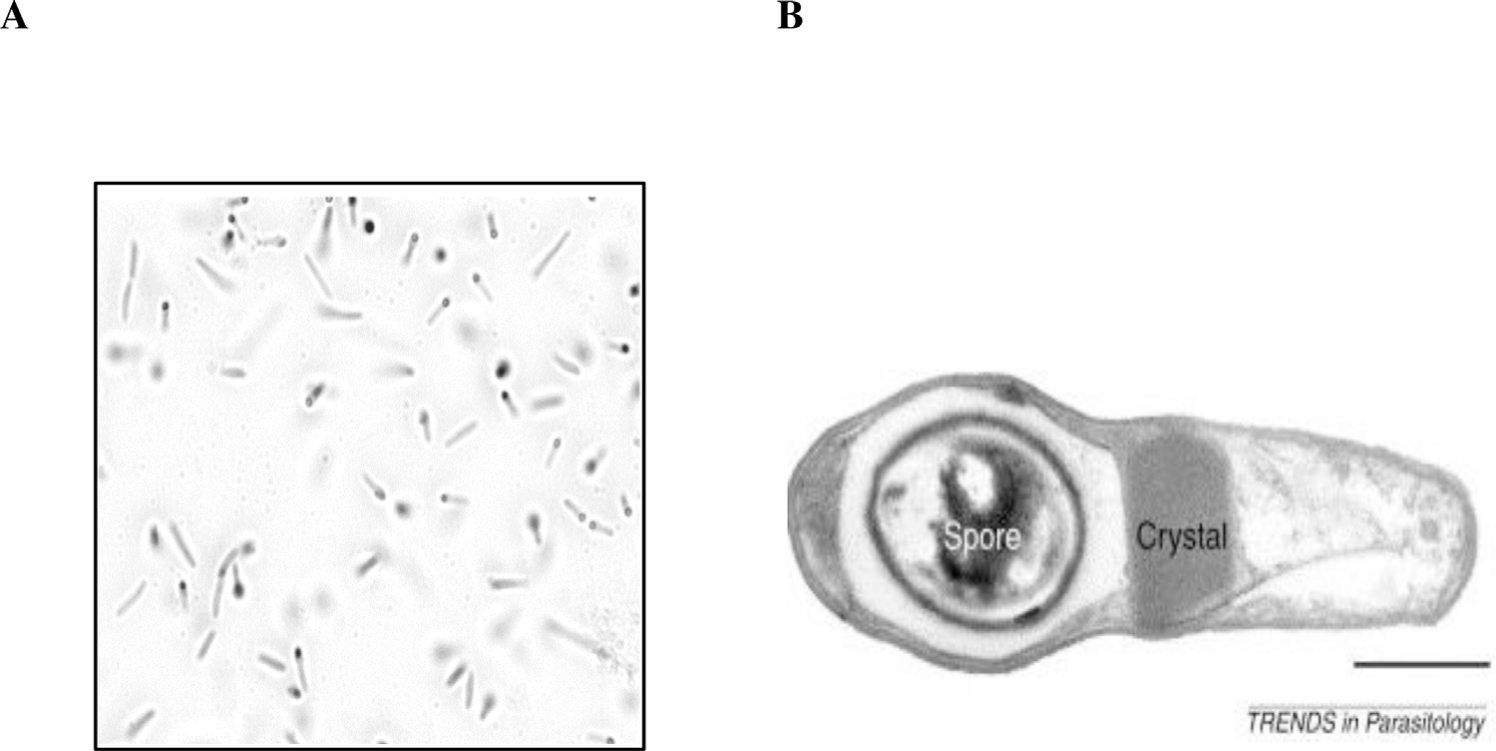
A) Sporulating *Lysinibacillus sphaericus* bacteria as seen under the microscope after malachite green staining. B) Electron micrograph showing the parasporal crystal of sporulated *L. sphaericus*(Regis, Silva-Filha, Nielsen-LeRoux and Charles, 2001). The parasporal crystal is composed of three proteins- BinA, BinB, and SlpC (Hire, Sharma, Hadapad and Kumar, 2014).

The infection cycle of Lsph begins with the uptake of bacterial spores by mosquito larvae. These spores are rich in crystals of BinAB proteins (Fig. 1). When ingested, the alkaline pH of the larval gut dissolves these parasporal crystals and releases two highly conserved protoxins (inactive forms): Pro-BinA (41.9 kDa) and Pro-BinB (51.4 kDa). The larval midgut proteases convert them into active forms. The protease activation of the toxin at alkaline-pH has been linked to the presence of pH-sensitive switches in BinA/B structures that facilitate spore-crystal dissolution in the larval midgut (Colletier et al., 2016). The active BinA (∼39 kDa) and BinB (∼42 kDa) proteins bind to their target site located on the brush border membranes of the midgut of the larval epithelial cells, and the toxin translocates further through the cell membrane. Inside the cell, the toxin induces cytopathological events such as vacuolization, autophagy, and apoptosis, which lead to larval death [Berry, 2012, Nielsen-Leroux and Charles, 1992, Opota et al., 2011]. The target receptor for BinAB is a glycosylphosphatidylinositol (GPI) - anchored protein, Cpm1/Cqm1, from Culex (Darboux, Nielsen-LeRoux, Charles and Pauron, 2001). The interaction between the receptor and BinAB proteins is crucial for intoxication. The lack of a membrane-bound receptor due to mutations in the *cqm*1/*cpm*1 gene makes the larvae insensitive to the binary toxin and is the main cause of resistance in the Culex population (Nielsen-Leroux, Charles, Thiery and Georghiou, 1995, Romão et al., 2006, Darboux et al., 2007).

The early steps of BinAB mediated toxicity—from ingestion of parasporal toxin crystals to their conversion into soluble active forms—are well established (Berry, 2012). Subsequent events, such as molecular details of receptor recognition by the toxin subunits, probable mechanism of toxin internalization via the midgut epithelial cell membrane, and cytopathological events that trigger larval death, are currently being investigated and discussed within the research community. Here we provide a comprehensive overview of the known and the recently experimentally determined data and proposals, and discuss our perspective on the likely mechanism of action of the binary toxin for *Culex quinquefasciatus*.

## Receptor recognition and assembly of BinAB proteins

The binary toxin consists of two main polypeptides –BinA and BinB. BinA is the toxic or the larvicidal component of binary toxin, and BinB is the receptor-binding component (Oei, Hindley and Berry, 1992). It is generally believed that the two components act synergistically and show maximum larvicidal activity at equimolar concentrations (Berry, 2012, Oei, Hindley and Berry, 1992, Broadwell, Baumann and Baumann, 1990, Limpanawat, Promdonkoy and Boonserm, 2009). But, BinA and PEGylated BinA—alone, without BinB—also show high larvicidal activity (Kale et al., 2013, Bideshi et al., 2017, Sharma et al., 2017).

**Figure 2:**
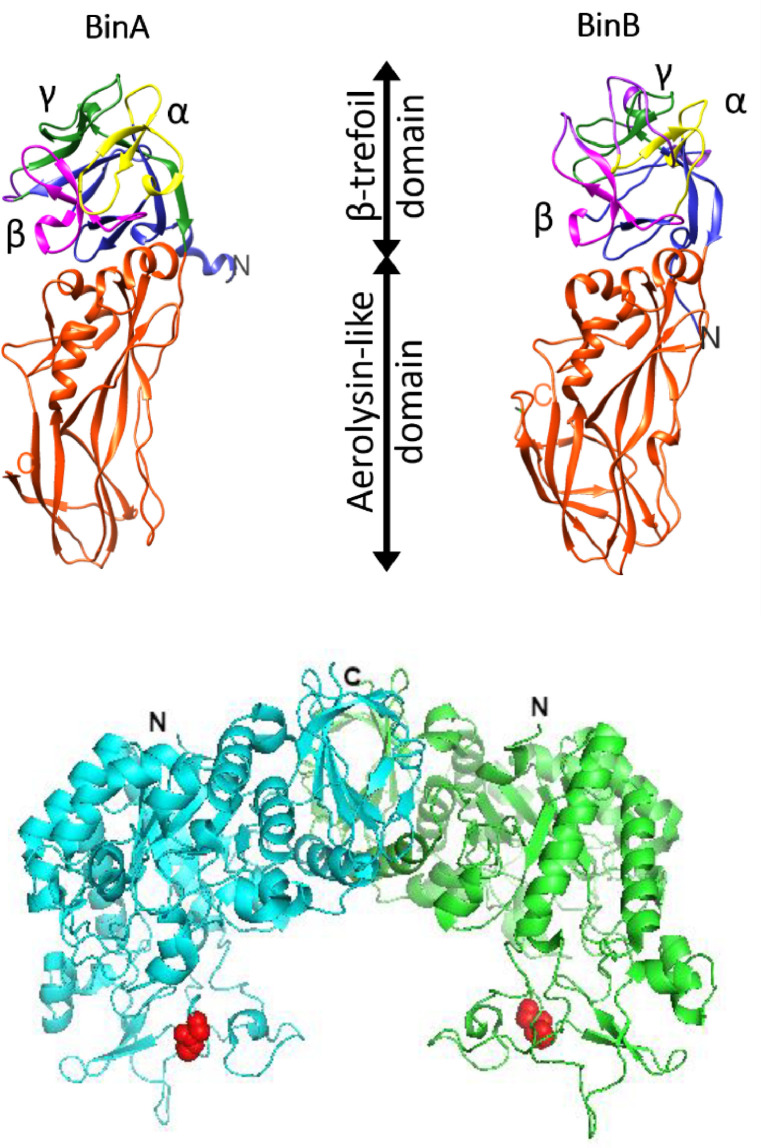
Ribbon models of A) BinA and BinB proteins with their N- and C- termini marked as N- and C- (Colletier et al., 2016). Both the proteins share a similar architecture and possess an N-terminal β-trefoil domain and an aerolysin-like C-terminal domain. B) Cqm1 protein (Sharma and Kumar, 2019). The protein exists as a dimer with the two monomers (shown in blue and green, respectively) held together weakly with solvation free energy gain of ∼4.4 kcal/mol on dimer formation. The 159GG160 residues believed to be crucial for BinB/Cqm1 interaction are shown as red spheres. Also marked are N- and C- termini of both the Cqm1 monomers.

The BinAB toxin binds to a specific receptor protein in Culex (Nielsen-Leroux and Charles, 1992). The receptor protein Cpm1/Cqm1 from *Culex pipiens* / *Culex quinquefasciatus* is an amylomaltase and belongs to the glycoside hydrolase family 13 subfamily 17 [GH 13_17] of the CAZy database (Darboux, Nielsen-LeRoux, Charles and Pauron, 2001, Sharma et al., 2018b). It is presented apically on the larval midgut epithelial membrane *via* a C-terminal GPI anchor (Nielsen-Leroux and Charles, 1992, Darboux, Nielsen-LeRoux, Charles and Pauron, 2001). The crystal structure of Cqm1 suggests that the protein in its native form is in a weakly held dimeric state (Sharma and Kumar, 2019) (Fig. 2B).

The specificity of the binary toxin is determined by its ability to recognize and bind to the target receptor in the larval midgut. Recently it has been suggested that affinity of BinA for some simple sugars and structurally different glycoproteins could also contribute towards receptor recognition (Sharma et al., 2018a). This understanding is primarily based on the seminal work of Srisucharitpanit *et al*. (Srisucharitpanit et al., 2014) and Colletier *et al*., (Colletier et al., 2016), who provided insights into the structures of the binary protein components. The first report on the crystal structure of the active BinB protein at a resolution of 1.75 Å by Srisucharitpanit *et al*. revealed two distinct structural domains for the BinB protein—the N-terminal β-trefoil domain and a C-terminal aerolysin-like domain. The β-trefoil scaffold is a highly conserved architecture of some carbohydrate-binding proteins, whereas the aerolysin domain occurs in many β pore-forming toxins. A detailed structural analysis of the parasporal crystals of BinAB by Colletier *et al*. (Colletier et al., 2016) showed that both BinB and BinA have similar N- and C- terminal domains (Fig. 2A). However, the β-trefoil scaffold of the BinA protein appears to be structurally capable of binding carbohydrates. In contrast, the pseudo-three-fold symmetry of the β-trefoil domain in the BinB protein is distorted due to loop insertion.

The binding of the BinAB toxin to its target receptor is a critical step for larvicidal activity. BinB protein facilitates the homing of BinA at the target site, owing to its high specificity binding (Sharma et al., 2018a). The sequence motif, 159GG160 doublet in the Cqm1 protein, is reported as crucial for binding the binary toxin (Ferreira et al., 2014). It was earlier proposed that the toxin components assemble onto the receptor as a heterotetrameric complex (Smith, Cámara-Artigas, Brune and Allen, 2005) and act synergistically to achieve maximum larvicidal activity at an equimolar ratio (Berry, 2012). However, a proteomics study revealed the presence of Pro-BinA, Pro-BinB, and SlpC proteins in the spore crystals without forming heteromeric complexes (Hire, Sharma, Hadapad and Kumar, 2014). Though a stable heterodimer of Pro-BinA and Pro-BinB proteins is anticipated from an extensive heterodimer interface contributed mainly by the pro-peptides of the two proteins in the crystal structure (Colletier et al., 2016). In contrast, trypsin activated (*in-vitro*) BinA and BinB proteins readily form heterodimers (Surya et al., 2016). Also, a preformed hetero-complex of BinA/BinB proteins displays marginally higher activity (Kale et al., 2013). However, recent studies have shown that BinA protein, or its modified form, such as PEGylated BinA, can carry out the larvicidal effect even without BinB (Kale et al., 2013, Bideshi et al., 2017, Sharma et al., 2017) (Table 1). Since the target specificity is not altered (Sharma et al., 2017), it is expected that BinA recognizes the same receptor and acts through it. BinA also shows an affinity for the Cqm1 polypeptide (*K*D, ∼2 µM), *albeit* weaker than BinB (*K*D, ∼10 nM) (Sharma et al., 2018b), and displays an affinity for some simple sugars and structurally diverse glycoproteins (Sharma et al., 2018a). Given the evidence, a dual avidity effect is proposed for the interaction of BinA with the Cqm1 polypeptide and the glycan core of its GPI anchor for receptor recognition by BinA [Sharma et al., 2018b, Sharma et al., 2018a]. Similar mechanisms for receptor recognition have been reported for other bacterial toxins such as aerolysin (Hong et al., 2002, Diep et al., 1998).

**Table 1:** LC_50_ values of recombinant BinA, PEGylated BinA, and BinAB mixture purified from *L. sphaericus* spore crystals, against 3rd instar *Culex quinquefasciatus* larvae.

**Protein**	**LC_50_ (ng/mL)**
BinA	21.1
PEGylated BinA	3.4
BinAB (mixture)	6.5

## Internalization of BinAB proteins

**Figure 3:**
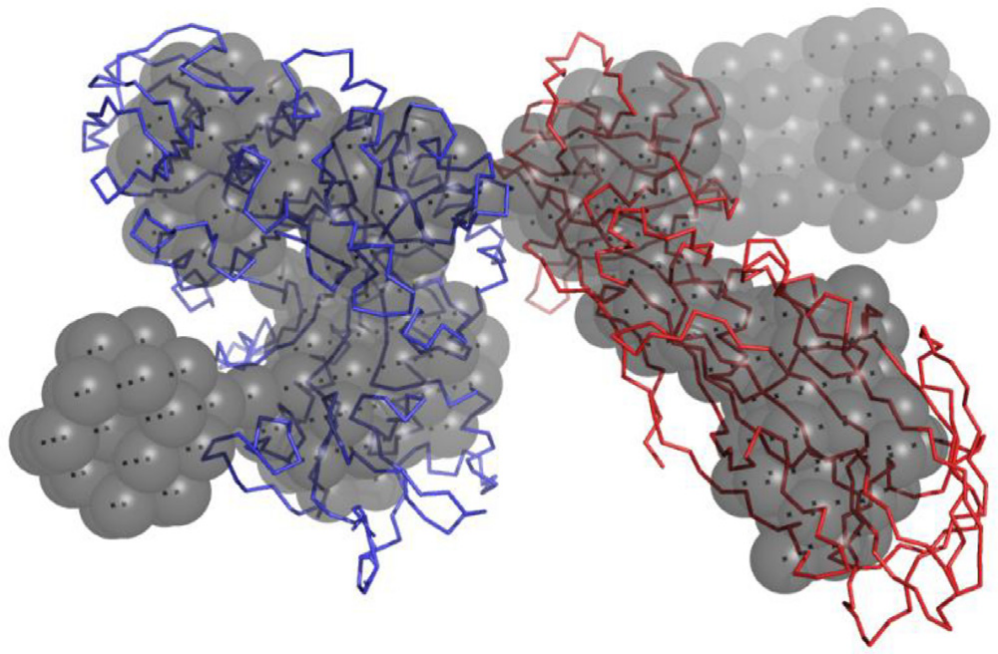
Low-resolution structural model of Cqm1-BinB complex derived from SANS studies. Binding of BinB changes oligomeric status of Cqm1 from dimer to monomer and heteromeric Cqm1–BinB complex structure (red ribbon, BinB; blue ribbon, Cqm1 monomer) is overlaid onto the *ab initio* shape models generated with DAMMIN (Svergun, 1999) (grey beads).

**Figure 4:**
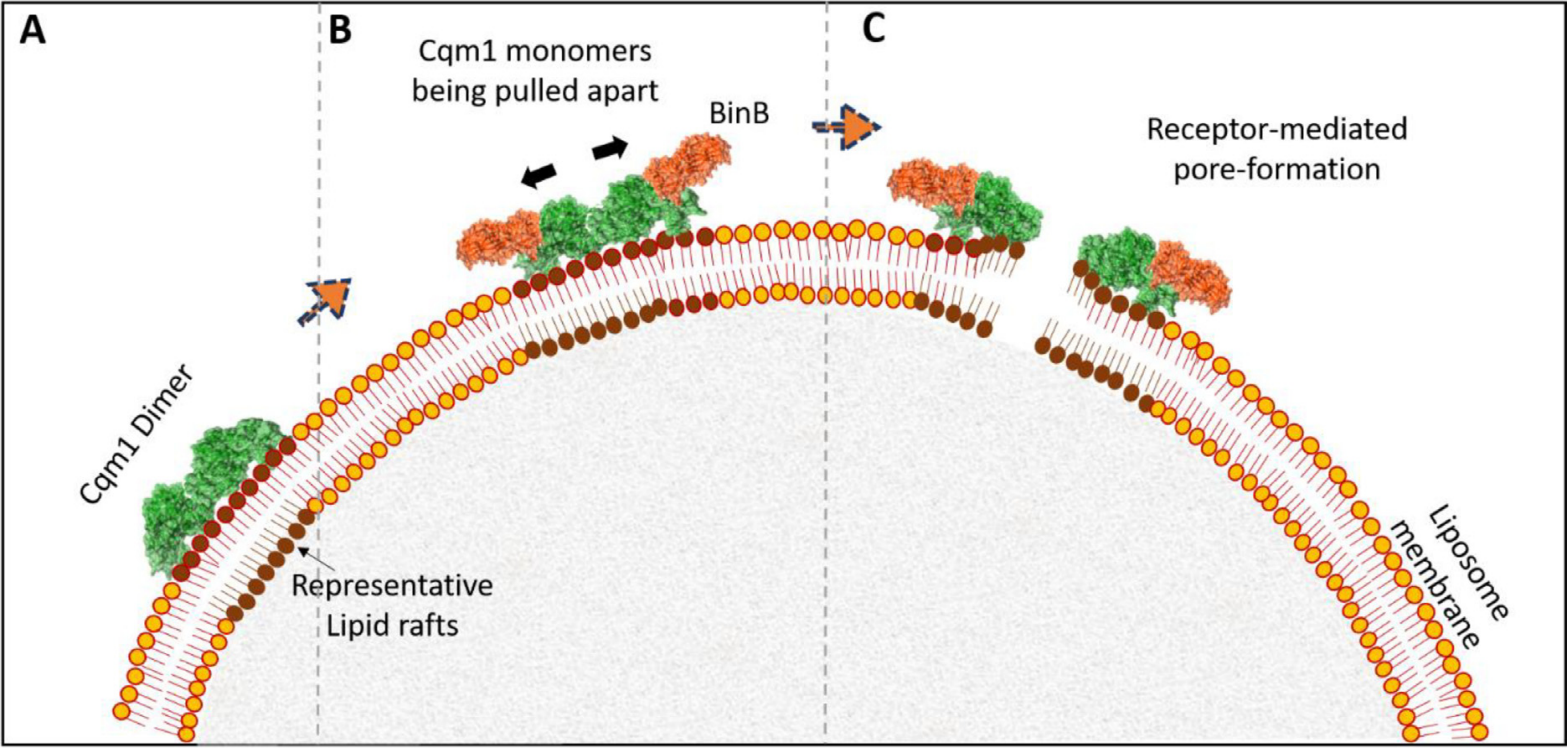
Proposed schematic of the receptor-dependent pore formation mechanism to internalize binary proteins across the Cqm1-loaded liposome membrane model. A) The ectodomains of Cqm1 dimer interact with the synthetic lipid-raft forming electrostatic contacts. B &C) BinB separates the weekly held monomers of the Cqm1 dimer, and this is shown here to cause a rupture in the dynamic structure of the lipid rafts.

The receptor Cqm1 protein from *C. quinquefasciatus* larvae mediates the translocation of the binary toxin across the cell membrane. Fluorescence-based studies confirmed the internalization of BinAB proteins and showed that the proteins are localized in the midgut epithelial cells of mosquito larvae (Lekakarn, Promdonkoy and Boonserm, 2015). However, the likely mode of toxin internalization has been a matter of debate. While some studies suggest a pore-forming model, similar to the *B. thuringiensis* δ-endotoxins (Vachon, Laprade and Schwartz, 2012, Bravo, Gill and Soberón, 2007), others impress upon receptor-mediated endocytosis. An important finding in this regard arose from the low-resolution solution structure of the BinB-Cqm1 complex, which was determined using ‘contrast-matched’ Small-Angle Neutron Scattering (SANS). It confirmed that the oligomeric state of Cqm1 protein changes from dimer to monomer upon binding to BinB (Sharma, Aswal, Gupta and Kumar, 2019, Sharma, Aswal, Kumar and Chidambaram, 2020) (Fig. 3). Based on our present model, we propose a schematic representation of the events involved in BinAB intoxication in Fig. 4.

In addition, the presence of electrostatic interactions between the ectodomain of Cqm1 and the lipid rafts reconstituted in liposome models, and the receptor-dependent impairment of the liposome models by the BinA/B proteins revealed a rupture of the artificial membrane (Sharma, Kumar and Kumar, 2020). The study also suggested that the receptor concentrated the toxin on the epithelial membrane. These observations indicate the internalization of the binary toxin by the receptor-dependent pore formation mechanism (Fig. 4). This mechanism seems plausible since lipid rafts are dynamic structures that rapidly assemble and disassemble again (Sezgin, Levental, Mayor and Eggeling, 2017). Typically, a rupture limit of ∼5-10 dynes/cm is required to form lipidic pores in the membrane (Evans, Heinrich, Ludwig and Rawicz, 2003). However, clathrin-dependent endocytosis has been suggested from a recent study with *A. gambiae* cell line (Riaz et al., 2020).

## Cytotoxicity by BinAB proteins

Cytopathological events such as vacuolation, autophagy, and apoptosis have been associated with BinAB mediated toxicity (Berry, 2012, Opota et al., 2011, Tangsongcharoen, Chomanee, Promdonkoy and Boonserm, 2015). The establishment of a reliable model system continues to provide critical information for the mode of action of the binary toxin. *Anopheles gambiae* Ag55 cell lines have been found to be very useful in this regard. The binary toxin was found to internalize and induce vacuolation in Ag55 cells expressing α-glucosidase gene (*Agm3*) (Hire et al., 2015). Pore-formation and vacuolation observed in binary toxin treated mammalian epithelial MDCK cell lines engineered to express the Cqm1 protein predict autophagy in response to binary toxin (Opota et al., 2011). Likewise, morphological changes such as mitochondrial swelling, chromatin condensation, and vacuolation, observed in Culex larvae fed with the binary toxin, point to apoptosis mediated by mitochondrial stress as a likely mechanism contributing to larval death (Tangsongcharoen, Chomanee, Promdonkoy and Boonserm, 2015). However, the actual mechanism by which the binary toxin exploits the cellular machinery to kill susceptible mosquito larvae has remained unclear. Interestingly, only a few studies suggest the release of cytochrome-c from mitochondria during ER-stress (Hetz, 2012), while others suggest a cytochrome-c independent activation of downstream apoptotic events in mammals in response to ER stress (Morishima et al., 2002). One of the more recent works suggests that BinA, due to its ability to bind certain carbohydrates, inhibits/disrupts the functioning of partially folded or functionally critical glycosylated proteins in the endoplasmic reticulum (Sharma et al., 2018a). This proposal relies on the observation that N-glycosylation is a critical post-translational modification for larval metamorphosis and development. When disrupted by mutations, chemical inhibitors, or RNAi, it turns out to be fatal (Walski et al., 2016). Interestingly, binary proteins have also been observed to affect mitochondrial respiration and cause cell death through apoptosis or autophagy [Riaz et al., 2020, Gupta et al., 2010].

Further, detailed transcriptome profiling of the susceptible and resistant strains of *C. quinquefasciatus* to the binary toxin has revealed many essential genes and differentially regulated pathways (Rezende et al., 2019). The resistant strains had Cqm1 as the most downregulated gene, resulting from a mutated gene, along with several enzymes involved in lipid catabolism. In contrast, enzymes involved in DNA synthesis and maintenance were highly upregulated. A similarly exciting observation was made by Riaz *et al*. (Riaz et al., 2020) through their studies on Ag55 cell lines, in which they found that BinAB proteins interacted primarily with the factors such as the clathrin heavy chain (endocytosis protein) and glycolysis enzymes such as pyruvate kinase, enolase, and dihydrolipoamide dehydrogenase. On the other hand, endocytic inhibitors such as Pitstop2 significantly reduced the viability of Bin-toxin-sensitive Ag55 cell lines. It will be interesting to see whether these differences in the responses of the cellular machinery of resistant and susceptible strains of Culex could be related to the events that lead to larval death.

## Challenges and future prospects

Lsph's BinAB proteins remain one of the most successful and highly specific insecticides with proven effectiveness. In this section, we mention some of the critical issues related to BinAB and highlight the recent advancements that could help overcome these challenges and extend a potential long-term impact. One of the main challenges to using BinAB in mosquito-control programs is the refractoriness of *Aedes aegypti* to the toxin—the reason for this remains mostly obscure. Proteins orthologous to Cqm1 in Anopheles (Agm3) and Aedes (Aam1) share almost 60% and 70% sequence identity with Cqm1, respectively (Ferreira, Romão, Pompílio de-Melo-Neto and Silva-Filha, 2010, Opota et al., 2008). Structural similarities between the orthologous of Cqm1 suggested that only a few amino acids may be critical for binding the binary toxin. These were later identified as the 159GG160 residues, which are conserved in Cqm1 and Agm3 but absent in Aam1, and could affect the binding of binary toxins to the Aam1 protein (Ferreira et al., 2014). Further, while the Cqm1 ortholog of Aedes (Aam1) displays α-glucosidase activity, Aam1 is glycosylated, but Cqm1 is not (Ferreira, Romão, Pompílio de-Melo-Neto and Silva-Filha, 2010). Once inside the cell, the BinA protein has been shown to cause mortality in *Ae. aegypti* (Bideshi et al., 2017). Whether the intrinsic high glycan affinity of BinA affects toxin internalization in *Ae. aegypti* thus requires further investigation and could pave the way for effective control of this mosquito vector. In addition, a consortium of vegetative Lsph cells has recently been found to be toxic to *Ae. Aegypti* (Rojas-Pinzón and Dussán, 2017). This would, however, require further investigation to assess the toxicity determinants involved and the consequences of their use in mosquito control.

Developing resistance among the mosquito population is another big challenge that compromises the long-term efficacy of the binary toxin as an effective mosquito larvicide. Binary toxin targets a single class of receptors inside the larval midgut (Nielsen-Leroux, Charles, Thiery and Georghiou, 1995). Although this imparts high specificity, it predisposes the toxin towards resistance. In contrast, mixtures of several δ-endotoxins in Bti parasporal crystals prevent the rise of resistance in the target species (Ben-Dov, 2014). As observed among the Culex population, the primary source of resistance towards BinAB has been the failure to present the receptor as a GPI-anchored protein on the midgut membrane. Mutations, deletions, or insertion of transposable elements reportedly cause the premature termination of the receptor polypeptide synthesis resulting in a truncated protein lacking the GPI anchor (Romão et al., 2006, Darboux et al., 2007, Chalegre et al., 2009, Guo et al., 2013). Therefore, targeted R&D efforts, like engineering the BinA/B proteins for an enhanced larvicidal activity or developing more potent bacterial strains with built-in mechanisms to prevent resistance, are required for effective integrated mosquito-control programs.

Chemical modifications such as the glutaraldehyde crosslinking of BinAB proteins *in vitro* (Kale et al., 2013) or the PEGylation of BinA (Sharma et al., 2017) have proven successful in improving their larvicidal activity against Culex without compromising the target specificity, making it safe. A chimeric bacterial construct expressing a mixture of insecticidal toxins BinA and Cyt1Aa from *L. sphaericus* and *B. thuringiensis subsp. Israelensis*, respectively, or a fusion protein consisting of Cyt1Aa and BinA polypeptides, has proven effective in managing binary-toxin resistant *C. quinquefasciatus* larvae and *A. aegypti* larvae which are not susceptible to the binary toxin (Nascimento et al., 2020, Bideshi et al., 2017, Federici, 2016). Cyt1Aa is a low-toxicity lipophilic protein that binds the midgut membrane and delays resistance. BinA and Cyt1A act synergistically and show larvicidal activity against a broad spectrum of mosquito species (Nascimento et al., 2020, Bideshi et al., 2017). Using such chemically improved proteins or a combination of toxin's active ingredients could help in broadening the spectrum of action for the toxin or lower the development of resistance among the target mosquito population.

In addition to their application in the biological control of mosquitoes, toxic Lsph strains have great potential for many other biotechnological applications. Comparative genome analysis of the central metabolism of Lsph showed that in addition to the insecticidal factors, toxic Lsph strains have a broad collection of genes related to traits such as toxic metal resistance and aromatic compound degradation (Gómez-Garzón, Hernández-Santana and Dussán, 2017). Due to its ability to bind metals (Lozano and Dussán, 2013, Edo and Dussán, 2016, Bustos, Ibarra and Dussán, 2018), Lsph has been the focus for the remediation of petroleum hydrocarbons (Pérez Rodríguez, Melo, Jiménez and Dussán, 2019, Hernández-Santana and Dussán, 2018, Manchola and Dussán, 2014) and biofilm formation (Narayanan, Choi and Han, 2019). The binary toxin is also being studied for its therapeutic role as a potential anti-cancer agent (Luo et al., 2014). Reportedly, the binary toxin induces apoptotic events in human cancer lines (Chankamngoen, Janvilisri, Promdonkoy and Boonserm, 2020) and targets the mitochondria (Kanwal, Abeysinghe, Srisaisup and Boonserm, 2021).

## Outlook

Since the discovery of bacterial strains with the potential for use in mosquito control programs, *L. sphaericus* has proven as a highly effective and environmentally-friendly approach. The binary (BinAB) toxin is primarily responsible for its larvicidal action and its acitivity is associated with the receptor Cqm1 protein.

Recent advancements in improvisation of the binary toxin for enhanced larvicidal effects, such as chemical modification of the BinA protein and the construction of chimeric proteins/bacterial strains with higher potency, have shown good promises. These have yet not been commercialized though. As shown by the detailed phylogenetic analysis, BinAB toxin can be considered safe for humans as Cqm1-like proteins are not observed in the human proteome (Pandey et al., 2021). The synergistic use of a combination of Bti and Lsph larvicides, however, needs to be carefully evaluated, given the recent alerts on the undesired effects of Bti on non-target organisms (Brühl et al., 2020) and there are not enough studies available to unequivocally conclude the safety of Cyt1Aa-BinA fusion towards non-target organisms. Further, compared to living organisms, the use of protein-based insecticides appears to be more environmentally friendly. The BinAB or the designed chimera proteins can be easily produced recombinantly and developed further into new molecules for better vector control.

Our understanding of the mechanism of toxin internalization, molecular events responsible for cytotoxicity inside the cell, and development of resistance in target species has improved in recent years. A better understanding of the mode of action for BinAB and other mosquitocidal proteins, and synergism between them, will help to develop effective biopesticides and facilitate overcoming the development of resistance in the future

## Author Contributions

**Mahima Sharma:** Writing – Original draft preparation, Visualization. **Vinay Kumar:** Writing – Reviewing and Editing, Supervision.

## Funding Source

No involvement of any funding source(s).

## CRediT authorship contribution statement

**Mahima Sharma:** Writing – original draft, Visualization. **Vinay Kumar:** Writing – review & editing, Supervision.

## Declaration of Competing Interest

The authors declare that they have no known competing financial interests or personal relationships that could have appeared to influence the work reported in this paper.
